# Sjögren’s Syndrome Associated With Erythema Dyschromicum Perstans: A Rare Dermatological Manifestation

**DOI:** 10.7759/cureus.84234

**Published:** 2025-05-16

**Authors:** Sonia Golani, Sulhera Khan, Zara Saeed, Humaira Talat, Nazish Shah

**Affiliations:** 1 Dermatology, Dow University of Health Sciences, Civil Hospital Karachi, Karachi, PAK; 2 Internal Medicine, Jinnah Postgraduate Medical Centre, Karachi, PAK; 3 Dermatology, Civil Hospital Karachi, Karachi, PAK; 4 Dermatology, Dr. Ruth K.M. Pfau Civil Hospital Karachi, Karachi, PAK

**Keywords:** ashy dermatosis, erythema cenicienta, erythema dyschromicum perstans, sjogren syndrome antibodies or ssa ssb, sjogren syndrome

## Abstract

Sjögren's syndrome is an autoimmune disorder with a complex, multifactorial etiopathogenesis that predominantly affects women, typically in their middle-aged years. The condition is associated with a variety of skin manifestations beyond the characteristic skin tightening and thickening. These include erythema multiforme, lichen planus, erythema nodosum (dermo-panniculitis), chilblain-like erythema, vasculitis, livedo reticularis, and granuloma annulare. One rare dermatological manifestation of Sjögren's syndrome is erythema dyschromicum perstans (EDP), also known as ashy dermatosis or dermatosis cenicienta, which is an acquired condition characterized by symmetrical hyperpigmentation on the trunk and extremities. Although few cases of ashy dermatosis have been reported in association with Sjögren's syndrome, we present the case of a 50-year-old woman diagnosed with Sjögren's syndrome and ashy dermatosis based on biopsy, marking what appears to be the first reported case from Pakistan.

## Introduction

Sjögren syndrome was coined by a Swedish physician, Henrik Sjögren, who first identified a group of women with arthritis associated with dry eyes and mouth [1]. It is a disorder of autoimmune origin with antibodies (antinuclear antibodies (ANA), present in over 80% of patients; rheumatoid factor (RF), positive in approximately 40%-60% of cases, and the more disease-specific anti-Sjögren syndrome-related antigen A (anti-Ro/SSA) and anti-Sjögren syndrome-related antigen B (anti-La/SSB) antibodies, which are present in 60%-75% and 40%-60% of patients, respectively), attacking the salivary and lacrimal glands, affecting the production of saliva and tears [2]. The exact pathogenesis of the disease appears to be multifactorial and is not completely understood [3]. Various theories have been proposed to explain the pathogenesis of Sjögren syndrome, including genetic susceptibility (HLA-DR and HLA-DQ alleles), environmental triggers (viral infections due to molecular mimicry), epithelial cell activation, immune dysregulation involving both innate and adaptive responses, and epigenetic modifications [3]. The disease can be primary or secondary and associated with other connective tissue disorders like rheumatoid arthritis (RA), systemic lupus erythematosus (SLE), and, less commonly, with multiple sclerosis, autoimmune hepatitis, and thyroiditis [2]. Apart from the dryness of the affected areas, commonly termed the sicca syndrome, the disease can present with systemic manifestations [3]. Systemic involvement most commonly involves the skin (23%-67%), followed by symmetric, non-erosive arthropathy (50%), muscle involvement as myalgias and muscle weakness (44%), renal involvement as distal renal tubular acidosis (RTA) (30%) followed by lymph nodes (10%), lungs (10%-20%), and brain (10%) [3].

The cutaneous features present as annular erythema or erythema multiforme, lichen planus or erythema nodosum (dermo-panniculitis), chilblain-like erythema, vasculitis commonly presenting as palpable purpura, cheilitis, xerosis, eyelid dermatitis, pruritus, livedo reticularis, and granuloma annulare [4]. Erythema dyschromicum perstans (EDP), also known as ashy dermatosis or dermatosis cenicienta, is a rare dermatological manifestation of Sjögren syndrome. It is an acquired disorder of hyperpigmentation, typically distributed symmetrically on the trunk and extremities [5]. The exact pathogenesis of ashy dermatoses is not entirely clear, but an association with autoimmune diseases, particularly autoimmune thyroiditis and Graves' disease, as well as vaccines (such as the influenza vaccine), has been identified [6]. Extensive ashy dermatosis, as in our patient, is believed to have an immune-mediated pathogenesis involving a T-cell-driven interface dermatitis targeting basal keratinocytes, leading to pigment incontinence, findings supported by histopathology in our patient. Its management includes immunomodulatory therapies such as topical or systemic corticosteroids, dapsone, clofazimine, and narrowband UVB phototherapy in refractory cases. We report one of the first cases after a thorough literature search of a 50-year-old woman diagnosed with Sjögren disease along with ashy dermatosis supported by a biopsy.

## Case presentation

**Figure 1:**
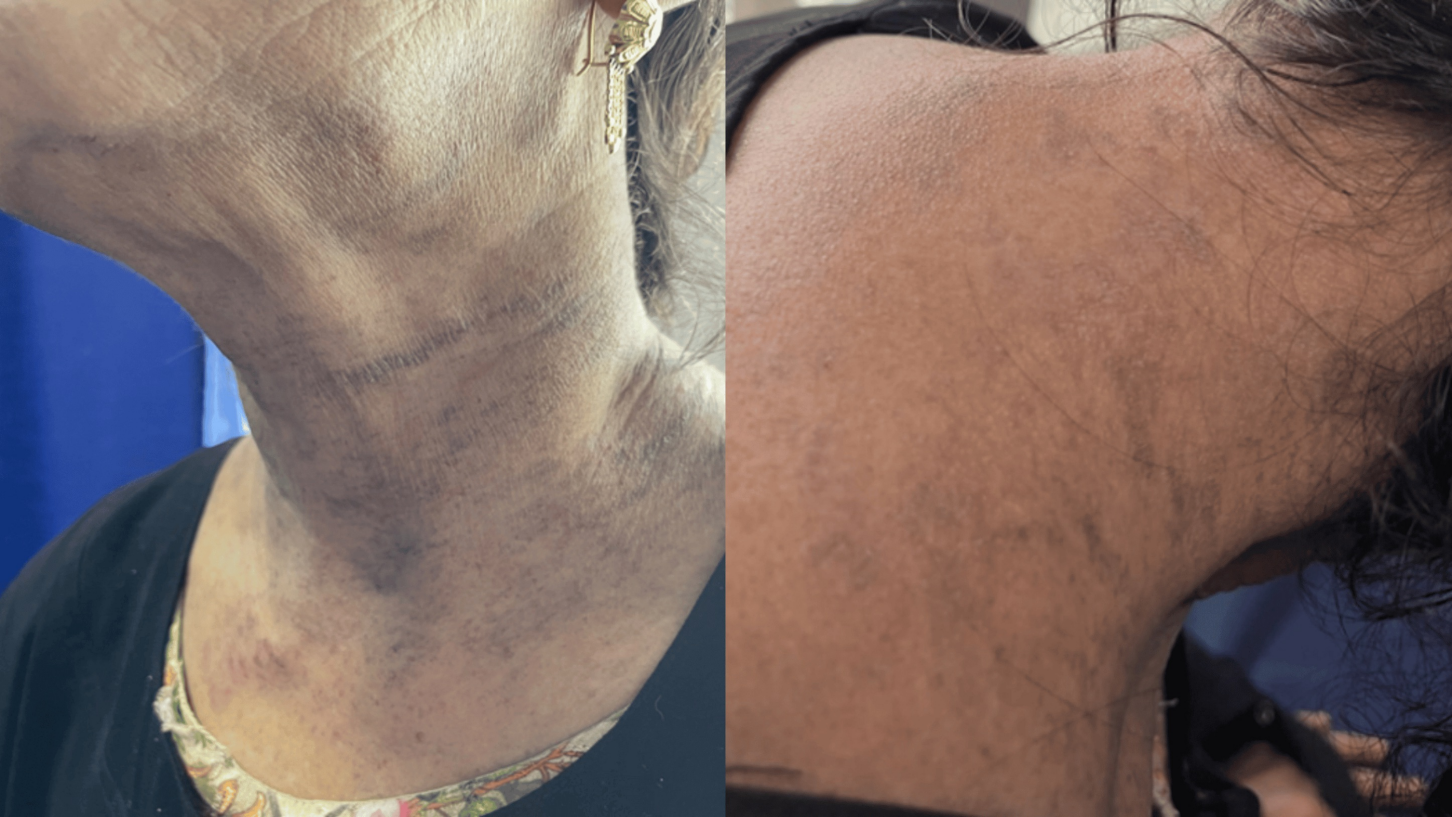
Hyperpigmented, violaceous linear macules and patches on the anterolateral and posterior aspects of the neck, consistent with EDP EDP: erythema dyschromicum perstans

**Figure 2:**
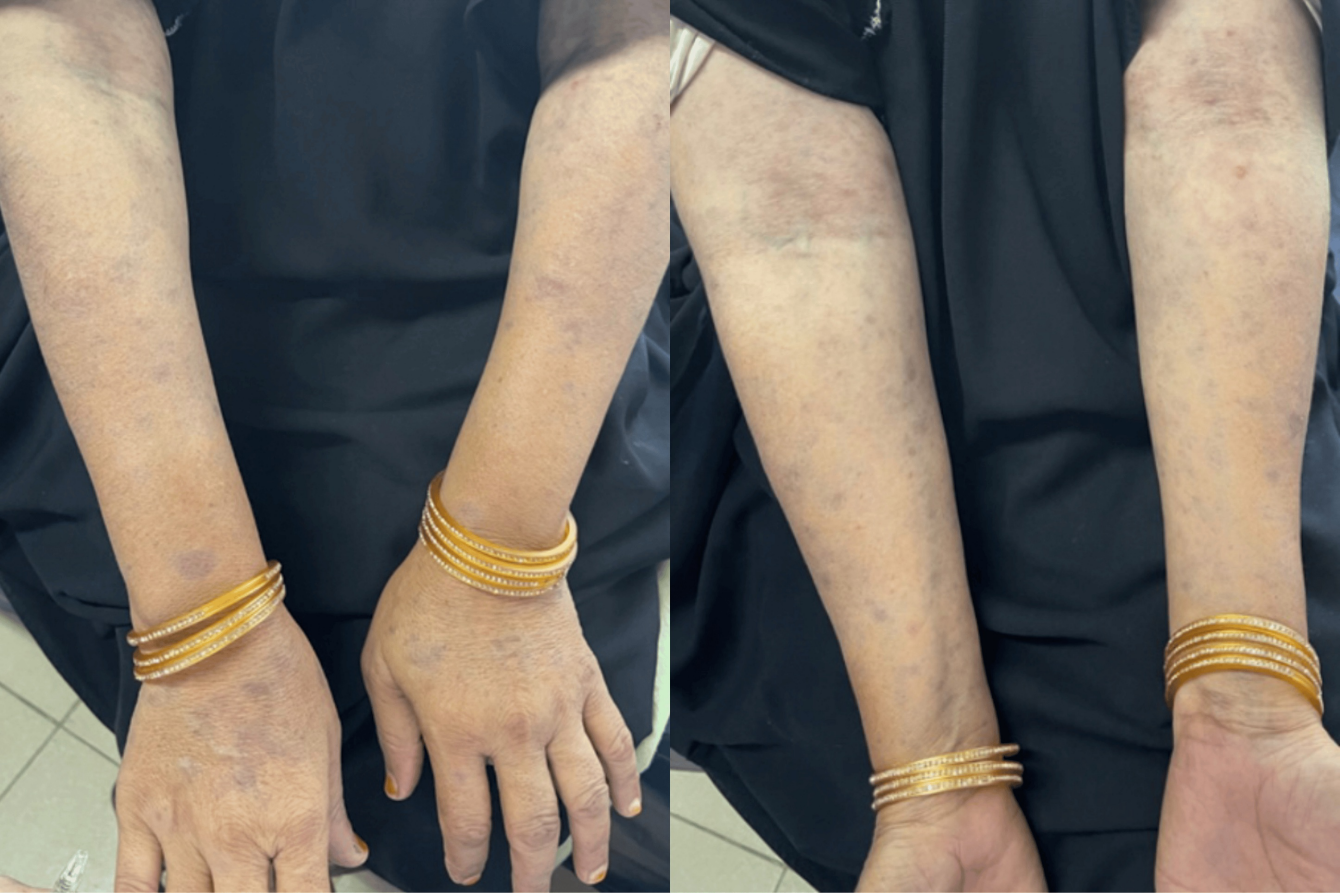
Violaceous, purplish macules and patches on the dorsal and ventral surfaces of the forearms and hands, consistent with EDP EDP: erythema dyschromicum perstans

**Figure 3:**
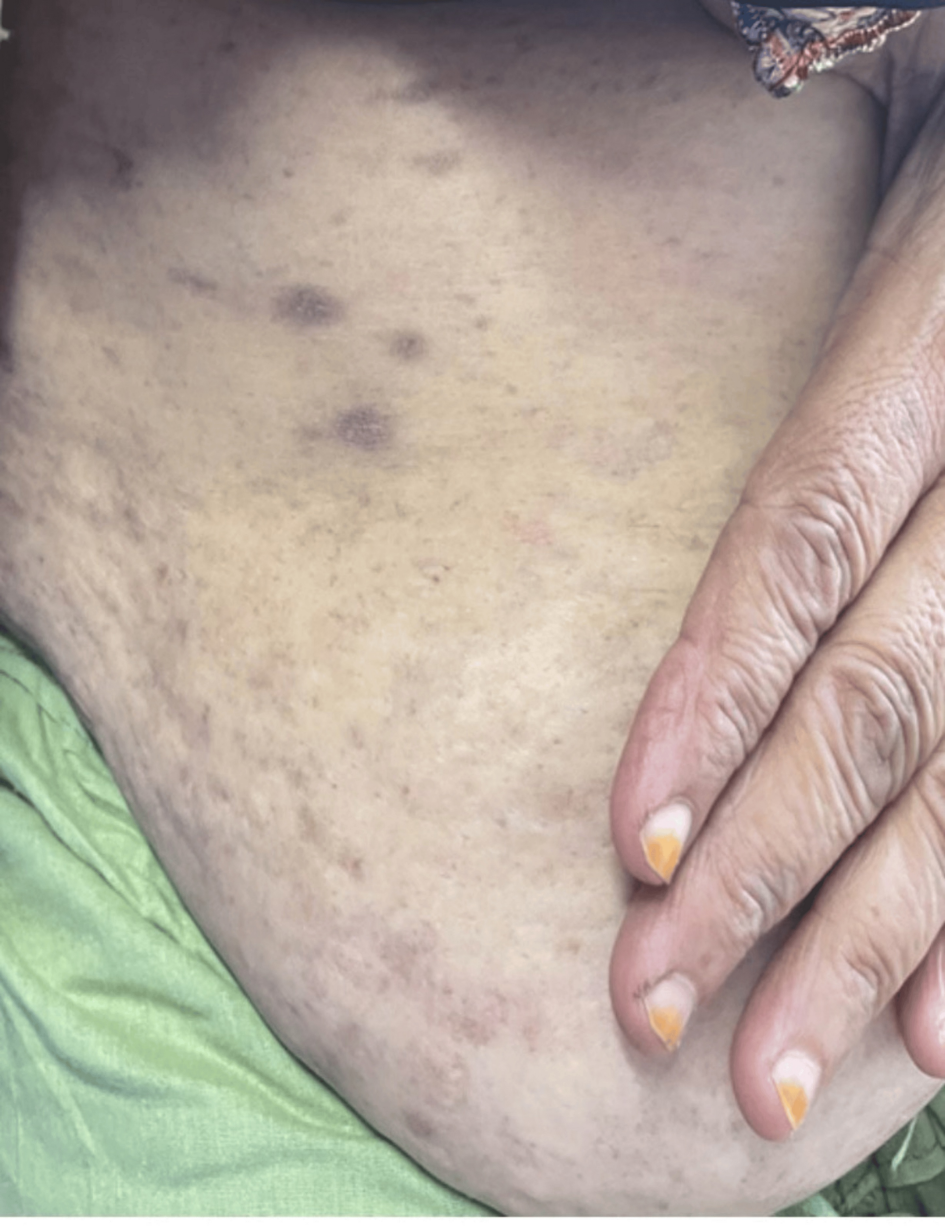
Violaceous, purplish macules on the abdomen

A 50-year-old female, resident of Karachi, Pakistan, with no known comorbidities, presented to the dermatology outpatient department of a tertiary care hospital with complaints of dry eyes and mouth for the last 2.5 years. She also complained of the development of pigmented gray to bluish-colored macules all over her body over the past two months. According to the patient, the dryness in her eyes progressed gradually over the course of time, leading to a complete absence of tear formation. The dryness of her mouth simultaneously progressed, causing the absence of saliva production. The dryness led to gritty eyes, foreign body sensation and irritation, and the dry mouth led to difficulty in chewing food, decreased appetite, weight loss, and recurrent oral infections. For the last two months, she had developed hyperpigmented, gray to light purplish-colored macular lesions of varying sizes involving the entire body and skin folds, sparing the palms, soles, and face as shown in Figures 1, 2, 3. On palpation, the skin was dry at a normal temperature.

They first began from the upper extremities and then progressed to involve the trunk and lower extremities. The lesions were pruritic but not painful. She denied the use of any drug prior to the onset of skin lesions. On the systemic review, there is a history of generalized arthralgia, reduced appetite, weight loss (undocumented), and photosensitivity. She denied any history of Raynaud’s phenomenon, fever, weight loss, oral ulcers, or alopecia. Sweating was absent on the whole body, even in the months of summer. Her past medical and family history is not significant.

On examination, she was vitally stable, well-oriented, and alert with a Glasgow Coma Scale (GCS) score of 15/15. Her sub-vital examination showed lymphadenopathy of the submental and anterior cervical lymph nodes on the right side. The nodes were non-matted, mobile, and non-adherent to the underlying or overlying structures. The systemic examination was unremarkable. The oral mucosa and tongue were dry and cracked without any oral ulcers. The lips had angular cheilitis. 

Her baseline laboratory investigations, including full blood count and liver and renal function tests, were normal. The inflammatory markers were raised with an erythrocyte sedimentation rate of 95 mm/hour (0-20 mm/hour); however, the C-reactive protein was normal. Her autoimmune profile revealed a positive anti-nuclear antibody (ANA), titers >1:160 with a speckled pattern (normal: <1:40), and positive anti-SSA (normal: <20U/mL) and anti-SSB autoantibodies (normal: <20 U/mL).

**Figure 4:**
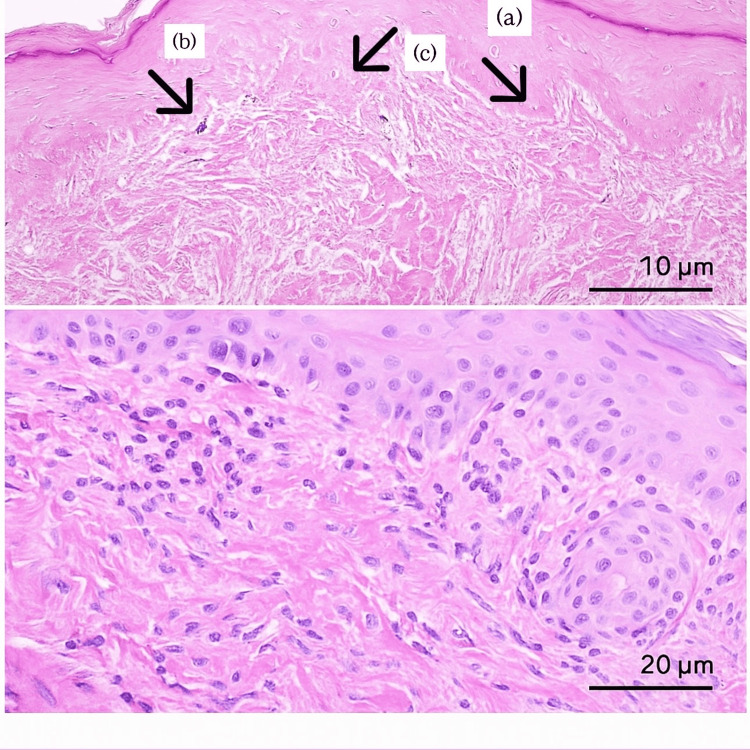
The overall architecture of the epidermis and dermis, including the undulating rete ridges (a), pigmentary incontinence (b), and a few colloid bodies (c) Histopathological examination of skin tissue (Hematoxylin & Eosin stain). (Above) Low-power view (10x objective). (Below) High-power view (40x objective). Scale bars: A=10 µm, B=20 µm.

A skin biopsy was performed on one of the hyperpigmented macules from the right arm, which showed a section of the intact epidermis with a preserved granular layer. There was perivascular lymphocytic infiltration in the superficial dermis, focally touching the interface. However, no saw-toothed appearance was seen. There was mild pigmentary incontinence along with a few colloid bodies (Figure 4). The above-mentioned features are non-specific and include the following differentials: lichen planus (characterized by lymphocytic dermal infiltrate; however, the absence of rete ridges argues against it), post-inflammatory hyperpigmentation (with pigment incontinence and dermal inflammation), and discoid lupus erythematosus (marked by epidermal atrophy, dermal infiltrate, and pigment incontinence). However, the findings can be diagnostic of EDP in the right clinical context, given the clinical picture of our patient. 

**Figure 5:**
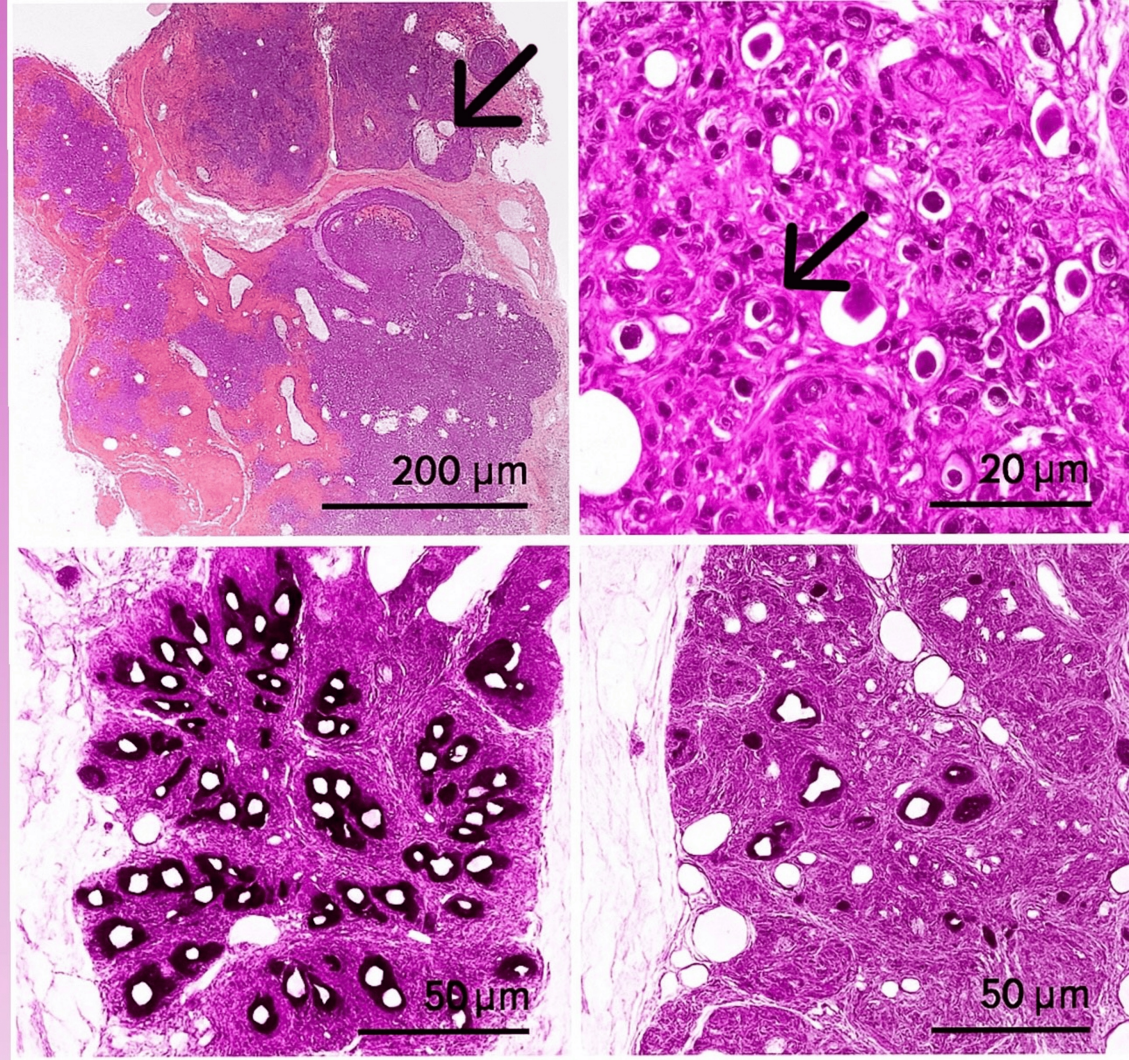
Sections from the minor salivary gland show mucinous acini with abundant foamy macrophages (top right), lymphoid aggregates in a background of interstitial fibrosis (top left), and mucinous acini with a few atrophied glands (bottom right and left images), suggestive of Sjögren's syndrome Histopathological examination of minor labial salivary gland tissue (Hematoxylin & Eosin stain). (Top right) Low-power view (4x objective). (Top left) High-power view (40x objective). (Below right and left) Intermediate magnification (10x-20x objective). Scale bars: top right=200 µm, top left=20 µm, below right and left=50 µm.

A labial mucosa biopsy was also performed, which showed minor salivary glands surrounded by moderate chronic lymphocytic infiltrate and lymphoid aggregates. Fibrosis was also noticed along with atrophy of the glands (Figure 5). The histopathological diagnostic criteria for Sjögren syndrome rely on the presence of focal lymphocytic sialadenitis (FLS) with a focus score ≥1 per 4 mm² of glandular tissue [7]. 

The clinical and histopathology findings are diagnostic of Sjögren syndrome. Hence, the diagnosis of EDP with Sjögren syndrome was made. The 2016 American College of Rheumatology (ACR)/European League Against Rheumatism (EULAR) Classification Criteria for Sjögren syndrome is a widely used standardized approach for diagnosing primary Sjögren's syndrome [7]. A total score ≥4 from the following weighted items in Table 1 confirms the diagnosis. Our patient met the criteria with a total score of 7 points; with a positive labial salivary gland histopathology (3 points), anti-SSA positivity (3 points), and a positive Schirimer's test (1 point).

**Table 1:** 2016 ACR/EULAR classification criteria for primary Sjögren’s syndrome ACR/EULAR: American College of Rheumatology/European League Against Rheumatism; SSA/Ro: Sjögren's-syndrome-related antigen A; OSS: ocular staining score

	Domain	Test	Score
1	Labial salivary gland biopsy	Focal lymphocytic sialadenitis with focus score ≥1/4 mm²	3
2	Anti-SSA/Ro antibody positivity	Detected via ELISA or immunoblot	3
3	OSS	OSS ≥5 (or van Bijsterveld score ≥4) in at least one eye	1
4	Schirmer’s test	≤5 mm/5 min in at least one eye	1
5	Unstimulated whole saliva flow	≤0.1 mL/min	1

An ophthalmological evaluation was conducted, and the Schirmer’s test showed reduced tear film production (<5 mm). A slit-lamp examination of the cornea and conjunctiva revealed no corneal ulcers or conjunctivitis. The patient was prescribed artificial tears and cyclosporine eye drops for the management of keratoconjunctivitis sicca and reduced tear production. To address the dry mouth, saliva substitutes (mouthwash and spray) were recommended, along with regular hydration and the use of sugar-free gum to stimulate saliva production.

For the arthralgias, a thorough rheumatological examination was performed, and the patient was started on non-steroidal anti-inflammatory drugs (NSAIDs) and hydroxychloroquine (HCQ) at a dose of 200 mg twice daily. A baseline electrocardiogram (ECG) was carried out to rule out potential cardiac issues related to the risk of QT prolongation, which came back normal.

The patient was advised on sun protection and the use of sunscreen to manage photosensitivity, as well as the application of topical emollients for skin dryness and tightening due to impaired sweating. The benign nature of EDP was explained, along with cosmetic measures such as camouflage creams. Treatment options, including topical corticosteroids, topical calcineurin inhibitors, narrowband UVB phototherapy, and pulsed-dye laser therapy, were discussed. However, due to the extensive nature of the lesions and the side effects of extensive steroid application, the decision was made not to proceed with topical corticosteroids or calcineurin inhibitors.

Narrowband UVB (NB-UVB) phototherapy was initiated for the hyperpigmentation, with two sessions per week for four weeks. However, minimal improvement in skin hyperpigmentation was observed, and the patient opted to discontinue further sessions.

The patient is currently on six-monthly follow-ups to assess the progression of Sjögren’s syndrome, monitor the skin lesions, and screen for any signs of systemic involvement.

## Discussion

EDP was first introduced by Ramirez (1957), who described the development of gray-ash-colored macules distributed symmetrically on the trunk and extremities of 139 patients in El Salvador who were labelled as ashy ones and the disease termed ashy dermatosis or dermatosis cenicienta [8]. Convit et. al reported five cases of similar dermatological manifestations in 1961 in Venezuela, which was then coined as EDP [9]. The disease is most common in people of Latin American descent [10].

The exact pathogenesis of the disease is still unknown. One suggested hypothesis is an abnormal immune response to antigens, characterized by a preponderance of CD8+ lymphocytes in the dermis, and HLA-DR+ intercellular adhesion molecule 1+ keratinocytes in the epidermis [8]. The major histocompatibility complex, specifically Human Leukocyte Antigen (HLA-DR4), is genetically associated with the disease [8].

The main etiology of the disease is not known. Most cases are idiopathic, but few associated factors have been identified, such as infections (intestinal parasites and human immunodeficiency virus (HIV)), cobalt allergy, radiocontrast media (barium sulfate), drugs (penicillin, benzodiazepines, ethambutol, fluoxetine, and omeprazole), herbal consumption (Tokishakuyakusa), endocrinopathies (hypothyroidism and diabetes mellitus), and dyslipidemia [11]. In our case, the associated etiology was Sjögren syndrome.

The classic dermatological presentation of EDP is the development of oval to round, gray, blue, or brown macules of various sizes, symmetrically distributed on the face, neck, trunk, and extremities [12]. A thin erythematous border may be found in the acute phase, which is characteristic of the disease [11]. The differentials of EDP include idiopathic eruptive macular pigmentation, lichen planus pigmentosus, maculopapular mastocytosis, postinflammatory hyperpigmentation, morphea, pityriasis rosea, Addison's disease, hemochromatosis, arsenism, contact dermatitis, multiple fixed drug eruption, and tuberculoid leprosy [5]. EDP can be differentiated from the differentials based on clinical examination and histopathology. Histopathology shows follicular hyperkeratosis, degeneration of the basal epidermis, and pigmentary incontinence with infiltration of melanin-carrying macrophages and histiocytes. The histopathologic changes seen in the skin are mainly in the papillary and subpapillary zones [9].

There are no published guidelines on the management of the disease. Some cases have undergone a spontaneous resolution, while in some cases the role of dapsone, clofazimine and ultraviolet (UV) phototherapy has been studied with positive results [9]. For cosmetic purposes, make-up and camouflage creams may be used. Our case however highlights one of the first presentation of ashy dermatoses with Sjögren syndrome in the literature.

The limitations of the study included its single-center design and the short follow-up period.

## Conclusions

Our case is one of the first in the literature to determine the association of EDP with Sjögren syndrome. The coexistence of the two disorders suggests that common immunological mechanisms may be responsible for pathogenesis. Therefore, it is imperative to consider that patients with Sjögren's syndrome may also present with a rare dermatological manifestation of EDP.
